# Nitrogen fertilization amplifies stem borer susceptibility in rice: genotype-dependent trade-offs between yield and resistance

**DOI:** 10.3389/fpls.2026.1789050

**Published:** 2026-06-26

**Authors:** Taha A. S., Amr Elkelish, Celestin Ukozehasi, AlShymaa Z. AL-Mokadem

**Affiliations:** 1 Rice Research Laboratory, Rice Research Department, Field Crops Research Institute, Agricultural Research Center, Cairo, Egypt; 2 Department of Biology, College of Science, Imam Mohammad Ibn Saud Islamic University (IMSIU), Riyadh, Saudi Arabia; 3 Department of Crop Science, School of Agriculture and Food Sciences, University of Rwanda, Kigali, Rwanda; 4 Chemistry Department, College of Science, Jouf University, Sakaka, Saudi Arabia; 5 Botany Department, Plant Physiology Laboratory, Women’s College, Ain Shams University, Cairo, Egypt

**Keywords:** biochemical traits, host plant resistance, nitrogen fertilization, rice (Oryza sativa), stem borer, yield

## Abstract

Rice (*Oryza sativa L*.) productivity is strongly influenced by nitrogen fertilization, genotype, and stem borer infestation. Understanding the interaction between nitrogen fertilization and genotype in determining host plant resistance and yield is essential for sustainable rice production. This study was conducted across the 2024 and 2025 growing seasons to evaluate the synergistic effects of rice genotypes and varying nitrogen application rates on insect infestation levels and agronomic performance. Four distinct genotypes were subjected to four nitrogen levels to assess their susceptibility to the rice stem borer, *Chilo agamemnon* Bles. Significant *(P* < 0.05) genotypic variation was observed regarding stem borer susceptibility. These results indicate that increasing nitrogen fertilization enhances grain yield while simultaneously increasing susceptibility to stem borer infestation, highlighting a potential trade-off between productivity and resistance. Across all genotypes, higher nitrogen applications (220 kg N/ha) significantly increased infestation percentages, while the lowest rates (55 kg N/ha) resulted in the least damage. Biochemical analysis revealed that the susceptible genotype (Super 302) possessed elevated concentrations of chlorophyll, total carbohydrates, and amines. In contrast, the more resistant genotypes (Giza 183 and GZ 10590) were characterized by significantly higher levels of protective phenols and silica. Regarding yield components, Giza 183 achieved the highest grain yields (averaging 11.03–11.06 t/ha), while Super 302 produced the lowest (9.88–9.93 t/ha). Although increased nitrogen rates up to 220 kg N/ha bolstered grain yields across all genotypes, they simultaneously exacerbated pest susceptibility. Notably, Giza 183 produced the highest number of panicles per hill at maximum N levels, whereas Super 302 was characterized by the earliest heading date and greatest plant height. These findings suggest that selecting for high silica and phenol content, combined with optimized nitrogen management, is critical for developing rice production systems that balance high productivity with robust insect resistance.

## 1 Introduction

Rice (*Oryza sativa* L.) stands as a foundational pillar of global food security, serving as the primary dietary staple for more than 50% of the world’s population (FAO, 2022; USDA Economic Research Service, 2023). Beyond its role as a source of calories, rice is a critical provider of essential nutrients, contributing approximately 15% of the global protein requirement and 21% of total energy intake for humanity (IRRI, 2023; Newilah et al., 2025; FAO, 2022). As the global population is projected to exceed nine billion by 2050 (United Nations, 2022, United Nations, 2022), the strategic management of rice production has become a focal point for international agricultural research (FAO, 2022; International Rice Research Institute, 2023). In the context of Egypt, rice is a vital cereal crop, yet its productivity is perennially challenged by an array of biotic and abiotic constraints (El-Ramady et al., 2022). Achieving the yield potential of modern rice varieties requires a sophisticated understanding of the complex interactions between genetic factors, environmental conditions, and agricultural practices (Kim and Kim, 2025). The quest to improve rice production is not merely about increasing raw tonnage but ensuring that such production is sustainable and resilient to the mounting pressures of climate change and evolving pest populations (Sarker et al., 2024).

Despite advancements in agricultural technology, insect pests continue to be a primary driver of crop loss in rice ecosystems worldwide (Deka et al., 2022). It is estimated that over 100 species of insects attack rice throughout its growth cycle, with at least 20 species capable of causing significant economic damage (IRRI, 2023; Chandrasiri et al., 2023). In Egypt, the entomological landscape is dominated by several key pests, including the rice stem borer (*Chilo agamemnon* Bles.), the rice leaf miner (*Hydrellia prosternalis* Deem.), and bloodworms (*Chironomus* spp.) (Mansour et al., 2022). Among these, the rice stem borer is particularly devastating, with recent research attributing approximately 6% of total crop losses to its activity (Soliman et al., 1997; El-Ramady et al., 2022). The damage caused by *C. agamemnon* is characterized by two distinct symptoms: “dead hearts” during the vegetative stage and “white heads” during the reproductive stage (Makkar and Bentur, 2017; Chandrasiri et al., 2023). These symptoms are the direct result of larval feeding within the stem, which severs the vascular tissues and prevents the transport of nutrients and water to the developing panicle (Chandrasiri et al., 2023). Because these larvae are protected within the plant tissue, chemical control is often difficult and inefficient, necessitating the development of alternative strategies such as host plant resistance (Subedi et al., 2024).

The development of high-yielding rice varieties with inherent resistance to biotic stresses is a primary objective for both rice breeders and entomologists (Haque et al., 2021; Hu et al., 2024). Host plant resistance (HPR) offers a multifaceted advantage: it reduces the farmer’s reliance on expensive chemical inputs, decreases the environmental footprint of rice cultivation, and provides a continuous, inherent form of insect control (Zheng et al., 2021; Horgan et al., 2023). Integrating resistant genotypes into Integrated Pest Management (IPM) systems is considered a cornerstone of eco-friendly agriculture (Desneux et al., 2022). Historical evaluations have demonstrated significant variability in resistance levels among different rice types (Horgan et al., 2021). For instance, *Indica and Indica/Japonica* crosses have frequently shown greater susceptibility to stem borer infestation than pure Japonica varieties (Shu et al., 2018; Horgan et al., 2021). This genetic variability provides a rich resource for breeding programs. However, before any promising line can be recommended for widespread cultivation, it must undergo rigorous Value for Cultivation and Use (VCU) assessments to ensure its performance across diverse environments and under various pest pressures (Dima et al., 2024).

Nitrogen (N) is arguably the most influential nutrient in rice cultivation, serving as a primary limiting factor for growth, development, and ultimate productivity (Govindasamy et al., 2023). It is central to most vital physiological processes, including the synthesis of chlorophyll, enzymes, and proteins that drive the plant’s metabolic machinery (Govindasamy et al., 2023). Consequently, the proper application and management of nitrogen fertilizers—including timing, rates, and application methods—are essential for maximizing nitrogen use efficiency (NUE) and grain yield (Wang et al., 2022; Liu et al., 2023). However, the intensive use of nitrogen fertilizers creates a physiological trade-off known as the “nitrogen-driven susceptibility” phenomenon (Zheng et al., 2021). Extensive research has documented a positive correlation between high nitrogen doses and increased susceptibility to insect pests across cropping systems (Chen et al., 2008; Zheng et al., 2021; Horgan et al., 2021). Excessive nitrogen often results in softer plant tissues and a more “succulent” host, which is highly attractive to herbivorous insects and facilitates larval survival and development (Rashid et al., 2023). This necessitates a delicate balancing act for growers: applying enough nitrogen to achieve high yields without inadvertently creating an environment that fosters pest outbreaks.

The interaction between rice plants and stem borers is mediated by the plant’s internal biochemical profile. Plant resistance is generally classified into two categories: constitutive (present regardless of pest presence) and induced (triggered by infestation) (Horgan et al., 2023). Biochemical studies of rice genotypes are critical for confirming the presence of physiological “antibiosis,” where specific plant constituents adversely affect the growth or survival of the pest (Javed et al., 2023). Key biochemical components involved in this defense-susceptibility axis include: Silica and Phenols: These act as primary defense mechanisms. High levels of silica provide a physical barrier that can wear down insect mandibles, while phenolic compounds act as chemical deterrents or toxins (Zhang et al., 2025). Varieties like Giza 183 and GZ 10590 have been noted for their higher concentrations of these compounds (Anis et al., 2022). Carbohydrates and Amines: These are often viewed as “susceptibility factors”. Insects are attracted to genotypes with high sugar and protein content, as these provide the necessary nutrients for rapid larval development. Susceptible genotypes, such as Super 302, typically harbor higher levels of total carbohydrates and amines. Chlorophyll: While essential for photosynthesis, higher chlorophyll levels are often associated with increased nitrogen content and plant vigor, which can paradoxically make the plant more visible and attractive to egg-laying adults (Zheng et al., 2021). The accumulation of these biochemical compounds determines the “resistance value” of a genotype (Javed et al., 2023). Understanding the correlation between these biochemical markers and infestation levels (dead hearts and white heads) is essential for developing predictive models for pest resistance (Javed et al., 2023). Given the complex interactions between nitrogen fertilization, plant biochemistry, and pest dynamics, this study was designed with a multi-dimensional approach (Zheng et al., 2021). The primary objective was to investigate the performance of four distinct rice genotypes (Giza 178, Super 302, Giza 183, and GZ 10590) in response to varying levels of nitrogen fertilizer (Govindasamy et al., 2023).

The rice cultivars, Giza 178, Super 302, and Giza 183, were selected as widely cultivated at Delta region, Egypt, and GZ 10590 was also included as a promising line, that will be released to rice growers in the very near future (RRTC, 2016). In addition, these genotypes have not been previously evaluated to rice stem borer infestations under variable levels of nitrogen.

The four genotypes selected for this study represent diverse genetic backgrounds within Egyptian rice germplasm. Giza 178 is an Indica/Japonica hybrid developed by the Egyptian Rice Research Program, characterized by high yield potential and moderate tolerance to common pests; it is widely cultivated across the Nile Delta region (RRTC, 2016). Super 302 is a Japonica variety recognized for high yield under favorable conditions, though it has been documented as susceptible to stem borer infestation due to its biochemical profile favoring herbivore nutrition (Anis et al., 2022). Giza 183 is an Indica/Japonica cross that has demonstrated consistent resistance to stem borers and stable high yield performance across multiple growing seasons, attributed to elevated silica and phenol concentrations (Anis et al., 2022). GZ 10590 is a promising Indica/Japonica breeding line currently under evaluation for commercial release, noted for its favorable biochemical resistance profile, including high silica deposition and phenolic compound accumulation (RRTC, 2016). To the authors’ knowledge, no prior studies have evaluated the susceptibility of these specific genotypes to rice stem borer infestation under variable nitrogen fertilization regimes.

The research specifically aimed to: 1) Quantify the susceptibility of these genotypes to the rice stem borer (*Chilo agamemnon* Bles.) and the rice leaf miner (*Hydrellia prosternalis* Deem.) under field conditions (Horgan et al., 2021); 2) Analyze the fluctuations in key biochemical constituents—including chlorophyll, silica, phenols, carbohydrates, and amines—as influenced by both genotype and nitrogen rate; 3) Evaluate the resulting impact on agronomic traits, grain yield, and yield components to identify genotypes that balance productivity with resistance. By elucidating these relationships, this study seeks to provide actionable insights for rice breeders and farmers, facilitating the development of sustainable production systems that utilize nitrogen more efficiently while minimizing the risk of devastating pest infestations (International Rice Research Institute, 2023). However, no previous studies have evaluated the interaction between nitrogen fertilization levels and stem borer infestation across these widely cultivated Egyptian rice genotypes under field conditions. The rice genotypes used in this study (Giza 178, Super 302, Giza 183, and GZ 10590) represent widely cultivated and promising varieties in the Egyptian Delta region, differing in agronomic performance and potential resistance to insect pests.

## 2 Materials and methods

### 2.1 Experimental site and environmental conditions

To evaluate the interaction between rice genotypes and nitrogen fertilization regimes, two field experiments were conducted during the 2024 and 2025 growing seasons. The research was hosted at the Experimental Farm of the Rice Research and Training Center (RRTC) in Sakha, Kafr El-Sheikh, Egypt. This location is representative of the primary rice-growing regions in the Nile Delta, providing an ideal environment for assessing both agronomic performance and natural insect pest pressure.

### 2.2 Genetic materials and trial design

The study evaluated four distinct rice genotypes characterized by varying genetic backgrounds and plant types. The specific parentage and origins of these genotypes are detailed in **Table 1**.

**Table 1:** Pedigree and characteristics of investigated rice genotypes.

Genotype	Parentage	Type	Origin
Giza 178	Giza 175 X/Milyang 49	Indica/Japonica	Egypt
Super 302	PTGMS-19 X JRL 26	Japonica	Egypt
Giza 183	Giza 178 X SKC23893	Indica/Japonica	Egypt
GZ 10590	GZ8126 X HR17570	Indica/Japonica	Egypt

Giza 178, Super 302, and Giza 183, were selected as widely cultivated at different regions of rice growing, and GZ 10590 was also included as a promising line, that will be released to rice growers in the very near future (RRTC, 2016). To the authors’ knowledge, no prior studies have evaluated, no investigations have been carried out to evaluate to rice stem borer infestations of these genotypes under variable levels of nitrogen. In addition, some of selected genotypes represent Japonica rice (Super 302), and some others represent Japonica/Indica rice (Giza 178 and Giza 183 GZ and 10590).

The experiments were laid out in a split-plot design with three replications. The main plots were assigned to the four rice genotypes, while the sub-plots were allocated to four nitrogen (N) fertilization levels: 55, 110, 165, and 220 kg N/ha. Each experimental plot measured 20 m² (4 m x 5 m).

### 2.3 Crop management and fertilization regimes

Seeds were sown at a rate of 144 kg/ha on May 2^nd^ in both seasons after being soaked for 24 hours and incubated for 48 hours to accelerate germination. Thirty-day-old seedlings were transplanted at a density of three seedlings per hill, with a spacing of 20 x 20 cm between hills and rows Christias et al. (1975).

The selected nitrogen levels (55, 110, 165, and 220 kg N/ha) represent low, recommended, and high fertilization regimes based on guidelines from the Rice Research and Training Center (RRTC, 2016). Nitrogen was applied in the form of urea (46% N) using a three-split application strategy, one-third was incorporated into the soil during puddling, as basal application, one-third 20 days after transplanting (DAT) as top-dressing, and one third 40 DAT as top-dressing.

Standard agricultural practices, including weed management via thiobercarb (Saturn 50% at 4L/ha) and irrigation, followed the official recommendations of the Rice Research and Training Center (RRTC, 2016).

### 2.4 Entomological assessments

The susceptibility of genotypes to the rice stem borer (*Chilo agamemnon* Bles.) was quantified through two primary indicators: dead heart percentage (DH%), calculated 50 DAT by examining five random hills per plot. The percentage was derived from the ratio of tillers exhibiting dead heart symptoms to the total number of tillers. White head percentage (WH%) was estimated three weeks prior to harvest using the same sampling method. WH% was determined by the ratio of tillers with white heads to the total number of tillers with panicles. No records were taken for numbers of larvae or pupae, as only dead hearts and white heads were considered for evaluating genotypic resistance.

Genotypes were categorized based on the RRTC standard evaluation system: Resistant (0–3%), Moderately Resistant (>3–6%), Moderately Susceptible (>6–9%), Susceptible (>9–12%), and Highly Susceptible (>12%) (RRTC, 2016).

### 2.5 Biochemical and physiological analysis

Biochemical constituents were determined at the heading stage to establish the physiological basis of resistance or susceptibility: Total Chlorophyll content was analyzed following the methods described by Wettstein (1957) and Fadeels (1962). Carbohydrates and Phenols were quantified in dried stem and leaf samples according to Williams (1984). Silica Content was determined in digested samples using the rapid determination method of Wei-min et al. (2005). Amino Acids/Amines were extracted from oven-dried leaves using 80% ethyl alcohol and quantified according to Christias et al. (1975). All chemical analyses were performed at the laboratories of the National Research Center, Dokki, Egypt. Pearson correlation coefficients were calculated to assess relationships between biochemical traits and infestation parameters (dead heart and white head percentages). Chlorophyll content was determined spectrophotometrically based on absorbance at specific wavelengths corresponding to chlorophyll a and b. Total carbohydrates were quantified using colorimetric methods based on sugar–reagent reactions. Phenolic compounds were measured using standard Folin-type assays reflecting total phenolic content. Silica content was determined following digestion and quantification of silicon-based residues. Total free amines were estimated based on colorimetric detection of amino compounds following extraction, expressed relative to dry weight.

### 2.6 Agronomic and yield data collection

At maturity, several traits were recorded, including days to 50% heading, plant height (cm), and the number of panicles per hill Horgan et al., 2023. Ten random panicles per plot were used to estimate panicle length, weight, and the number of filled grains Hu et al., 2024. Grain yield was determined by harvesting a central 6 m² area of each plot, with final weights adjusted to a standard 14% moisture content.

### 2.7 Statistical analysis

Data were analyzed using a split-plot analysis of variance (ANOVA), where genotype was assigned to the main plots and nitrogen levels to the sub-plots. Analyses were conducted separately for each growing season. Prior to analysis, data were checked for normality and homogeneity of variance. Means were compared using Duncan’s Multiple Range Test (Duncan, 1955) at P ≤ 0.05. Pearson correlation analysis was performed to evaluate relationships between biochemical traits and infestation parameters. All statistical analyses were conducted using COSTAT software. Data from the two seasons were analyzed separately and are presented independently.

## 3 Results

### 3.1 Susceptibility of rice genotypes to rice stem borer infestation

**Figure 1:**
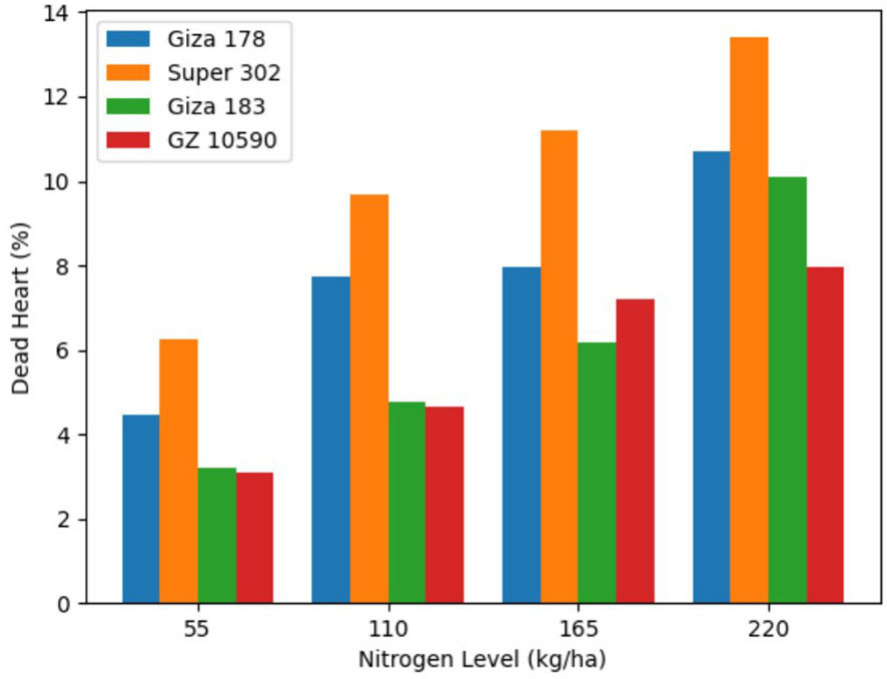
Effect of nitrogen fertilization levels on rice stem borer infestation, expressed as dead heart (%) and white head (%) across two growing seasons. Values represent seasonal means.

The susceptibility of the evaluated rice genotypes to the rice stem borer (*Chilo agamemnon* Bles.) differed significantly among each other in both 2024 and 2025 growing seasons, the genotypes fell into the resistance category across both seasons (**Figure 1**).

Genotypic variations.

The variety Super 302 exhibited the highest average infestation, recording 10.51% dead hearts and 9.74% white heads, which led to its classification as a susceptible (S) genotype. In contrast, Giza 183 variety and GZ 10590 promising line demonstrated significantly lower infestation levels, with mean dead heart percentages of 5.53% and 5.72%, respectively, categorizing them as moderately resistant (MR). Giza 178 variety displayed intermediate levels of infestation (7.82% dead heart and 6.85% white head on average) and was categorized as moderately susceptible (MS).

Nitrogen fertilization significantly influenced infestation levels. Nitrogen fertilization rates significantly impacted infestation severity across all genotypes. The highest borer infestation occurred at 220 kg N/ha level (averaging 10.48% dead hearts and 9.35% white heads), while the lowest incidence was observed at the 55 kg N/ha level. As nitrogen levels increased from 55 to 220 kg N/ha, the mean white head percentage increased by 142.9%.

Interaction Effects: Significant interactions between rice genotypes and nitrogen fertilization levels were observed for both dead heart and white head symptoms. As shown in **Table 2**, the combination of Super 302 and 220 kg N/ha resulted in the maximum levels of dead hearts (13.40% in 2024 and 14.57% in 2025) and white heads (13.00% in 2024 and 13.67% in 2025). Conversely, the lowest infestation levels were consistently recorded in the GZ 10590 promising line when fertilized with only 55 kg N/ha.

**Table 2:** Rice stem borer infestation as affected by the interaction between genotypes and nitrogen levels.

Treatment	2024				2025			
N kg/ha	55	110	165	220	55	110	165	220
Dead hearts %								
Giza 178	4.45 f	7.75 d	7.97 d	10.70bc	4.90 hi	5.10 g-i	10.10 c	11.60 b
Super 302	6.25 e	9.67 c	11.20 b	13.40 a	7.33 ef	9.40 cd	12.33 b	14.57 a
Giza 183	3.20 g	4.75 f	6.18 e	10.11bc	2.43 j	3.65 ij	6.38 fg	7.35 ef
GZ 10590	3.10 g	4.67 f	7.20 de	7.95 d	3.85 ij	4.45 i	6.30 f-g	8.15 de
White heads %								
Giza 178	3.80fg	7.50b-d	7.20cd	6.90c-e	4.40 gh	4.85f-h	9.95c	10.20c
Super 302	5.03d-g	10.20 b	9.60bc	13.00a	5.77fg	9.13cd	11.55b	13.67a
Giza 183	4.23e-g	5.0d-g	7.10cd	9.10bc	1.73j	2.30j	4.65gh	7.76e
GZ 10590	2.85g	2.63 g	5.97d-f	6.03d-f	2.93ij	3.93hi	6.20f	8.20de

Means within a column followed by the same letter do not differ significantly.

(*P* < 0.05) according to Duncan’s Multiple Range Test.

### 3.2 Biochemical composition of rice genotypes

The biochemical constituents of the rice plants varied significantly based on both genotype and the level of nitrogenous fertilization applied.

**Figure 2:**
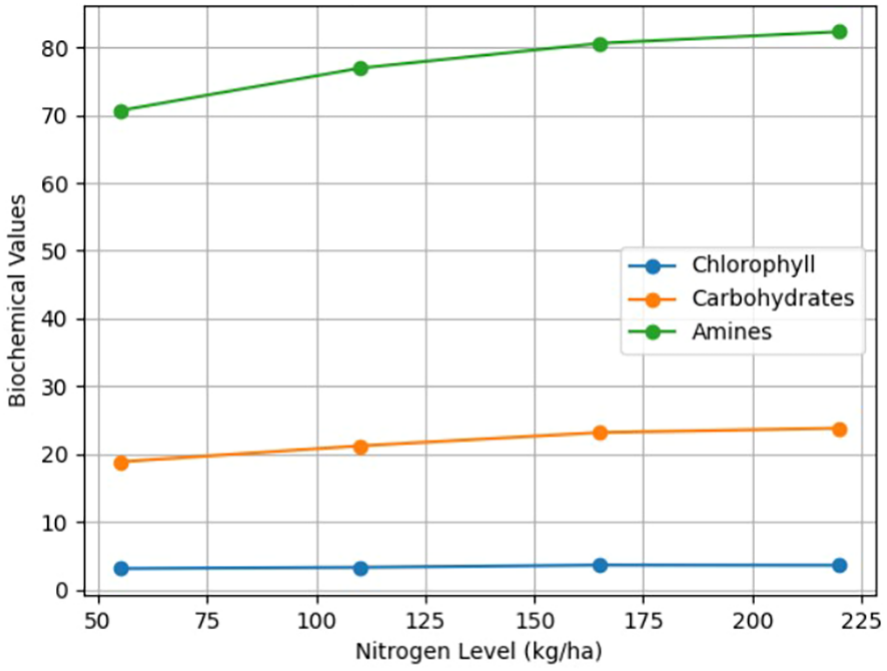
Variation in key biochemical traits (chlorophyll, total carbohydrates, and Total free amines) in response to increasing nitrogen fertilization levels. Values represent mean responses across genotypes.

Super 302 recorded the highest levels of total chlorophyll, total carbohydrates, and amines, while simultaneously possessing the lowest levels of phenols and silica compared to the other genotypes. Giza 183 and GZ 10590 were characterized by the highest concentrations of silica and total phenols. Detailed biochemical profiles for each genotype are presented in **Figure 2**.

Generally, the application of higher nitrogen doses enhanced the concentration of all biochemical components. The 220 kg N/ha rate yielded the highest values for chlorophyll, silica, phenols, carbohydrates, and amines, whereas the 55 kg N/ha rate yielded the lowest.

The interaction between genotypes and nitrogen levels, detailed in **Table 3**, revealed that chlorophyll a reached its peak in Super 302 at 220 kg N/ha. Silica and phenol levels were highest in GZ 10590 at the maximum nitrogen level, while the highest carbohydrate and amine concentrations were detected in Super 302 at the same rate.

**Table 3:** Analysis of some biochemical constituents of rice genotypes as affected by the interaction between genotypes and nitrogen levels.

Treatment	2024				2025			
	55	110	165	220	55	110	165	220
Chl a								
Giza 178	3.17h	3.31g	3.67b	3.61cd	3.27i	3.43g	3.76de	3.71ef
Super 302	3.45e	3.60cd	3.79a	3.78a	3.41g	3.67f	3.93a	3.87b
Giza 183	2.87j	3.15h	3.63c	3.59d	2.95k	3.27i	3.78cd	3.83bc
GZ 10590	2.90j	3.05i	3.37f	3.34fg	3.06j	3.35h	3.81c	3.75de
Silica % in stems								
Giza 178	3.24 g	3.31 f	3.49 d	3.43 e	3.18 l	3.29 j	3.41 g	3.37 h
Super 302	3.05 i	3.17 h	3.31 f	3.23 g	3.07 n	3.14 m	3.29 j	3.24 k
Giza 183	3.42 e	3.51 d	3.63 c	3.61 c	3.34 i	3.47 e	3.57 c	3.53 d
GZ 10590	3.53 d	3.65 b	3.73 a	3.75 a	3.45 f	3.59 c	3.67 b	3.71 e
Phenols								
Giza 178	10.75l	12.67j	15.35f	16.24e	11.51k	13.24j	15.48f	17.19d
Super 302	9.69m	11.28k	14.84gh	15.12fg	9.85l	11.33k	15.41fg	15.21g
Giza 183	13.35i	14.64h	16.92d	17.68c	13.71h	15.22g	18.62b	18.19 c
GZ 10590	13.58i	14.45h	18.69b	19.73a	13.47i	16.35e	19.63a	19.78a
Total carbohydrate								
Giza 178	19.67i	20.55h	22.95d	23.77c	19.85h	21.70f	24.35c	24.81c
Super 302	19.87i	22.33e	25.71 b	26.46a	20.50g	23.26d	27.35a	26.73b
Giza 183	18.63j	21.19g	22.35e	22.78d	18.70i	21.57f	22.85de	23.10d
GZ 10590	17.23k	20.71h	21.65f	22.21e	17.97j	20.90g	22.50e	23.17d
Total free amines (mg g-1 DW)								
Giza 178	67.59l	73.40i	77.50g	79.72ef	68.70ij	74.80h	79.10e	81.40d
Super 302	75.6h	82.60c	85.20b	87.37a	76.80g	83.70c	86.50b	89.700a
Giza 183	69.03k	75.50h	78.80f	80.70de	67.90j	74.70h	78.43f	80.10e
GZ 10590	70.40j	76.20h	80.90de	81.45cd	69.80i	77.40g	81.30d	82.90c

Means within a column followed by the same letter do not differ significantly.

(*P* < 0.05) according to Duncan’s Multiple Range Test.

### 3.3 Relationship between biochemical traits and stem borer infestation

**Figure 3:**
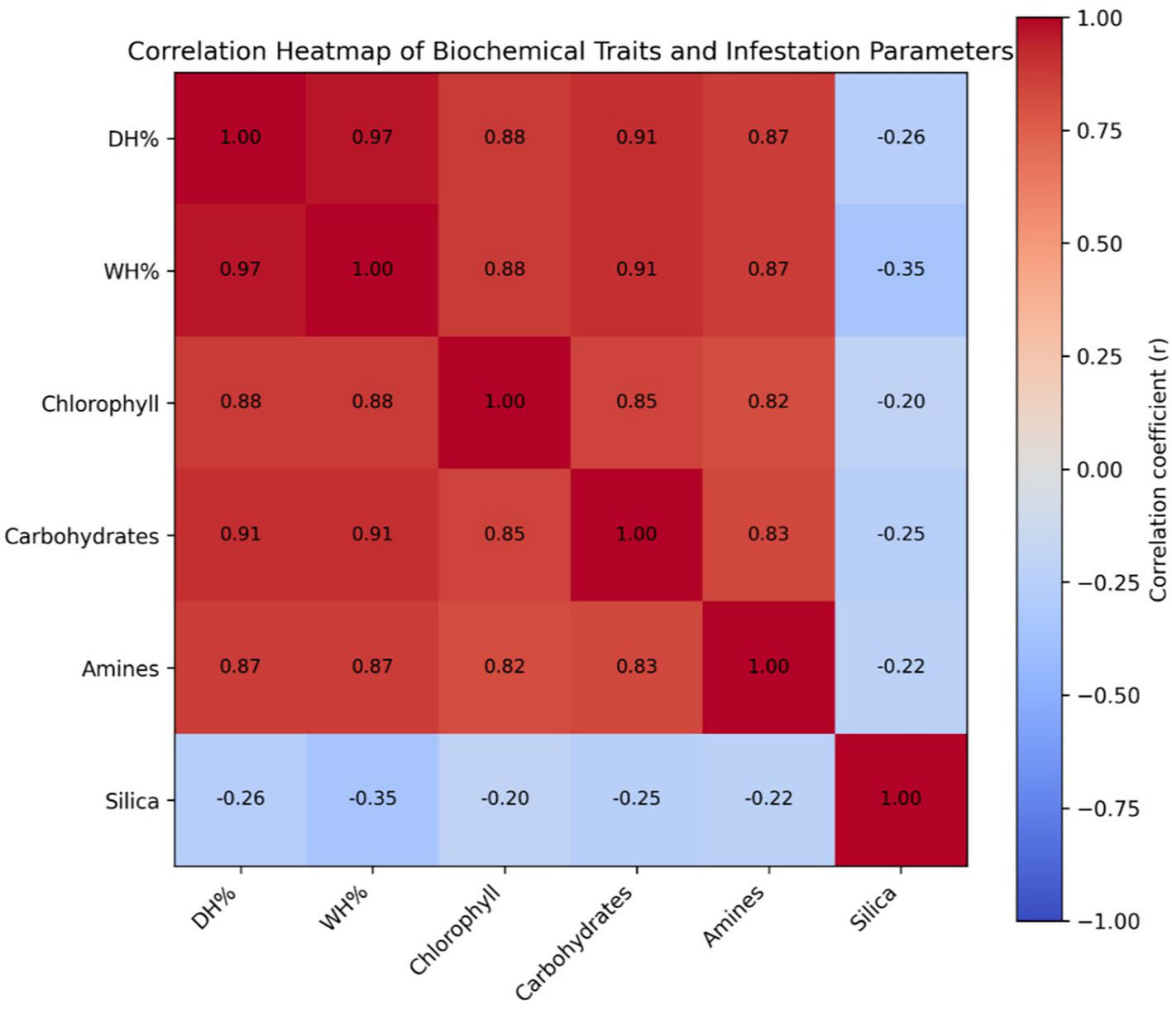
Correlation matrix showing relationships between biochemical traits and stem borer infestation parameters (DH% and WH%).

Pearson correlation analysis revealed significant relationships between biochemical traits and stem borer infestation parameters (Fig. X). Dead heart and white head percentages were positively correlated with total chlorophyll, carbohydrates, and amines, indicating increased susceptibility with higher nutritional content. In contrast, silica and total phenols showed negative correlations with infestation parameters, suggesting their role in enhancing plant resistance. Significant positive correlations were found between dead heart percentage and white head percentage (r = 0.967), total chlorophyll (r = 0.8806), total carbohydrates (r = 0.9123), and total amines (r = 0.8686). In contrast, silica content showed a negative correlation with both dead heart percentage (r = -0.2607) and white head percentage (r = -0.3468). These correlation coefficients are displayed in **Figure 3**.

### 3.4 Agronomic performance and yield attributes

Agronomic traits were significantly influenced by both genotype and nitrogen fertilization levels (**Tables 4**, **5**). Significant genotypic variation was observed across all measured traits. Giza 183 exhibited superior agronomic performance, recording the highest grain yield (11.03–11.06 t/ha) and the greatest number of panicles per hill. In contrast, Super 302 produced the lowest grain yield despite having the highest panicle weight and plant height. GZ 10590 and Giza 178 showed intermediate performance across most traits.

**Table 4:** Effects of rice genotype and nitrogen fertilization levels on agronomic traits and grain yield of rice (*Oryza sativa L*.) across two growing seasons.

Treatment	Days to 50% heading	Plant height (cm)	Panicles/hill	Panicle weight (g)	1000-grain weight (g)	Filled grains/panicle	Grain yield (t/ha)
Genotype							
Giza 178	100.25 a	99.95 b	21.53 b	3.58 c	20.64 d	156.0 b	10.74 b
Super 302	90.90 c	108.55 a	17.55 c	6.08 a	25.38 b	210.2 a	9.91 d
Giza 183	92.05 b	94.30 c	25.63 a	3.80 b	26.47 a	138.2 c	11.05 a
GZ 10590	92.00 b	91.15 d	25.36 a	3.76 b	25.18 c	132.1 d	10.56 c
F-test	P ≤ 0.05	P ≤ 0.05	P ≤ 0.05	P ≤ 0.05	P ≤ 0.05	P ≤ 0.05	P ≤ 0.05
Nitrogen (kg/ha)							
55	91.50 d	88.80 d	18.45 d	3.72 d	24.75 a	135.1 d	8.56 d
110	93.00 c	96.00 c	21.90 c	4.12 c	24.56 b	154.9 c	10.07 c
165	94.20 b	101.90 b	24.27 b	4.52 b	24.31 c	170.8 b	11.66 b
220	96.55 a	107.25 a	25.49 a	4.85 a	24.03 d	175.7 a	11.94 a
F-test	P ≤ 0.05	P ≤ 0.05	P ≤ 0.05	P ≤ 0.05	P ≤ 0.05	P ≤ 0.05	P ≤ 0.05

Values represent means of two growing seasons.

Means within each column followed by the same letter are not significantly different according to Duncan’s Multiple Range Test (DMRT) at P ≤ 0.05.

Genotype (G) and nitrogen level (N) effects were tested using split-plot ANOVA.

Values represent mean responses of each factor. Means within each column followed by the same letter are not significantly different according to Duncan’s Multiple Range Test (P ≤ 0.05).

**Table 5:** Interaction effect of rice genotype and nitrogen fertilization level on selected agronomic traits and grain yield.

Genotype	N (kg/ha)	Plant height (cm)	Panicles/hill	Grain yield (t/ha)
Giza 178	55	93.7	17.58	8.70
Giza 178	220	107.2	24.20	12.17
Super 302	55	97.3	15.25	8.05
Super 302	220	115.7	20.10	11.11
Giza 183	55	83.8	20.43	9.04
Giza 183	220	104.3	28.57	12.30
GZ 10590	55	81.7	20.63	8.51
GZ 10590	220	101.6	28.20	12.13

Values represent mean responses under each treatment combination.

Only selected nitrogen levels (minimum and maximum) are presented to illustrate interaction trends. The genotype × nitrogen interaction was statistically significant (P ≤ 0.05).

Values represent mean responses under each treatment combination. The genotype × nitrogen interaction was statistically significant (P ≤ 0.05).

Nitrogen fertilization significantly enhanced plant growth and productivity. Increasing nitrogen levels from 55 to 220 kg N/ha resulted in progressive increases in plant height, panicle number, and grain yield. The highest nitrogen level (220 kg N/ha) produced maximum grain yield and growth performance, while the lowest level (55 kg N/ha) resulted in the lowest values across all parameters. The interaction between genotype and nitrogen level was significant for key agronomic traits, indicating differential genotypic responses to nitrogen fertilization. Giza 183 showed the greatest yield response under high nitrogen conditions, achieving the highest grain yield at 220 kg N/ha. Although Super 302 exhibited substantial increases in plant height and panicle weight with increasing nitrogen, its grain yield remained comparatively lower than other genotypes. Similarly, GZ 10590 maintained stable performance with moderate yield increases under higher nitrogen levels. Overall, these results demonstrate that nitrogen fertilization enhances agronomic performance; however, the magnitude of response is strongly dependent on genotype.

## 4 Discussion

The present study provides a comprehensive evaluation of the interactive effects of genotype and nitrogen fertilization on rice stem borer infestation, biochemical composition, and agronomic performance. The findings clearly demonstrate that both genetic background and nitrogen availability play decisive roles in shaping plant susceptibility and productivity, with a pronounced interaction between these factors. Importantly, the results reveal a consistent pattern in which increased nitrogen fertilization enhances crop performance while simultaneously elevating vulnerability to stem borer infestation, reinforcing the concept of a physiological trade-off between growth and defense.

Significant genotypic variation in susceptibility to *Chilo agamemnon* was observed, with Super 302 consistently exhibiting the highest levels of infestation, whereas Giza 183 and GZ 10590 displayed relatively greater resistance. These differences can be attributed primarily to inherent biochemical composition, which constitutes a central determinant of host plant resistance (HPR) (Horgan et al., 2021; Javed et al., 2023). The susceptible genotype Super 302 was characterized by elevated concentrations of chlorophyll, total carbohydrates, and free amines, all of which are associated with enhanced nutritional quality for herbivorous insects. In contrast, the more resistant genotypes accumulated higher levels of silica and phenolic compounds, which function as structural and chemical defenses (Zhang et al., 2025).

These findings strongly support the concept of constitutive resistance, whereby baseline biochemical traits determine the degree of plant susceptibility independently of pest attack (Horgan et al., 2023). High silica deposition strengthens plant tissues and increases abrasiveness, thereby impairing larval feeding efficiency, while phenolic compounds exert toxic or deterrent effects through oxidative stress and enzyme inhibition (Zhang et al., 2025). The observed negative correlation between silica content and infestation parameters further confirms its protective role. Conversely, elevated carbohydrate and amine levels in susceptible genotypes enhance larval growth and survival by providing readily available energy and nitrogen sources.

Nitrogen fertilization emerged as a dominant factor influencing both plant performance and pest dynamics. Increasing nitrogen levels significantly increased infestation rates across all genotypes, with the highest damage consistently recorded at 220 kg N/ha. This pattern aligns with the widely recognized “nitrogen-driven susceptibility” hypothesis, which posits that excessive nitrogen fertilization promotes plant traits that favor herbivore development (Zheng et al., 2021). In the present study, higher nitrogen levels were associated with increased chlorophyll content, carbohydrate accumulation, and amine concentration, all of which contribute to enhanced host attractiveness and suitability.

Mechanistically, nitrogen plays a central role in plant metabolism, particularly in the synthesis of proteins, enzymes, and chlorophyll, thereby enhancing photosynthetic capacity and biomass production (Govindasamy et al., 2023). However, this metabolic stimulation comes at the expense of defensive investment. According to the carbon–nutrient balance (CNB) theory, increased nitrogen availability shifts resource allocation toward growth-related processes while reducing the synthesis of carbon-based defensive compounds (Herms and Mattson, 1992; Bryant et al., 1983; Zheng et al., 2021). This trade-off was clearly evident in the present study, where high nitrogen treatments resulted in increased susceptibility despite improved growth performance.

The role of amines in mediating plant–insect interactions is particularly noteworthy. Elevated amine levels under high nitrogen conditions provide a rich nitrogen source for insect larvae, facilitating rapid development and increased survival rates (Zhang et al., 2025). Recent studies have demonstrated that herbivore attack can induce polyamine biosynthesis pathways, particularly through the activation of arginine decarboxylase genes, leading to increased accumulation of compounds such as putrescine (Zhang et al., 2025). These compounds not only serve as nutritional resources but may also interfere with plant defense signaling pathways, including jasmonic acid-mediated responses, thereby weakening plant resistance.

In addition to biochemical changes, nitrogen-induced alterations in plant morphology and physiology contribute to increased susceptibility. High nitrogen availability promotes rapid cell division and elongation, resulting in softer, more succulent tissues that are easier for larvae to penetrate (Rashid et al., 2023). This structural vulnerability is further exacerbated by reduced silica accumulation under excessive nitrogen conditions, as silicon uptake is often negatively affected by high nitrogen supply (Zhang et al., 2025). The combined effect of enhanced nutritional quality and reduced physical defense creates an optimal environment for stem borer infestation.

The interaction between genotype and nitrogen level provides critical insights into the differential responses of rice varieties to fertilization regimes. While all genotypes exhibited increased infestation under high nitrogen conditions, the magnitude of this effect varied significantly. Giza 183 demonstrated a more balanced response, maintaining relatively lower infestation levels while achieving high grain yield, indicating a favorable combination of productivity and resistance. In contrast, Super 302 showed a pronounced increase in susceptibility with increasing nitrogen, suggesting that its genetic background predisposes it to nitrogen-induced vulnerability.

These findings highlight the importance of genotype selection in optimizing nitrogen use efficiency and pest management. Resistant genotypes with higher baseline levels of silica and phenols are better equipped to withstand the negative effects of excessive nitrogen fertilization. This observation is consistent with previous studies emphasizing the role of genetic variability in determining plant response to both nutrient availability and pest pressure (Hu et al., 2024). The integration of such genotypes into breeding programs represents a promising strategy for developing resilient rice varieties that combine high yield potential with enhanced resistance.

The agronomic results further underscore the complex relationship between nitrogen fertilization, plant growth, and pest susceptibility. Increasing nitrogen levels significantly improved yield components, including plant height, panicle number, and grain yield, with the highest values recorded at 220 kg N/ha. However, these gains were accompanied by increased infestation levels, particularly in susceptible genotypes. This dual effect illustrates the inherent trade-off between maximizing productivity and maintaining plant resistance.

The positive correlations observed between infestation parameters and chlorophyll, carbohydrates, and amines provide further evidence of the role of plant nutritional status in determining susceptibility. In contrast, the negative correlation between silica and infestation highlights the importance of structural defenses in limiting pest damage. These relationships are consistent with previous studies demonstrating that plant biochemical composition is a key determinant of host suitability for insect pests (Javed et al., 2023).

The findings of this study have important implications for sustainable rice production. While nitrogen fertilization is essential for achieving high yields, excessive application can compromise plant resistance and increase reliance on chemical pest control. Therefore, optimizing nitrogen management is critical for balancing productivity and pest resistance. Strategies such as split nitrogen application, use of controlled-release fertilizers, and precision nutrient management can help minimize excessive nitrogen accumulation and reduce pest risk (Sajjad et al., 2024; Wang et al., 2022).

Despite the strengths of this study, certain limitations should be acknowledged. The absence of uninfested control treatments and temporal biochemical measurements restricts the ability to distinguish between constitutive and inducible resistance mechanisms. Future research should incorporate controlled infestation experiments and time-course analyses to better understand the dynamics of plant defense responses. Additionally, molecular and genomic approaches—including genome-wide association mapping of the loci that govern antioxidant and biochemical defenses—could resolve the genetic architecture underlying the genotype-specific responses observed here, as demonstrated for antioxidant-defense and nutrient-related traits in cereals (Alqudah et al., 2025a, 2025b). A further open question is whether a genotype’s defensive biochemical state is fixed within a single generation or can be primed and transmitted across generations; emerging evidence for intergenerational and transgenerational stress memory in cereals (Elkelish et al., 2025; Thabet et al., 2024, 2025) indicates that defensive investment may be modulated across generations, a promising avenue to integrate into host-plant-resistance breeding against stem borer.

## 5 Conclusion

In conclusion, the results of this study clearly demonstrate that nitrogen fertilization and genotype interact to determine rice susceptibility to stem borer infestation. High nitrogen levels enhance plant growth and yield but simultaneously increase susceptibility through biochemical and physiological changes. Genotypes with higher levels of silica and phenolic compounds exhibit greater resistance, highlighting the importance of biochemical traits in host plant defense. These findings emphasize the need for integrated strategies that combine optimized nitrogen management with the use of resistant genotypes to achieve sustainable rice production systems.

## Data Availability

The original contributions presented in the study are included in the article/supplementary material. Further inquiries can be directed to the corresponding authors.
